# Intentions and experiences of effective practice in mental health specific supported accommodation services: a qualitative interview study

**DOI:** 10.1186/s12913-017-2411-0

**Published:** 2017-07-11

**Authors:** Sima Sandhu, Stefan Priebe, Gerard Leavey, Isobel Harrison, Joanna Krotofil, Peter McPherson, Sarah Dowling, Maurice Arbuthnott, Sarah Curtis, Michael King, Geoff Shepherd, Helen Killaspy

**Affiliations:** 1 Unit for Social and Community Psychiatry, WHO Collaborative Centre for Mental Health Services Development, Queen Mary University of London, Newham Centre for Mental Health, London, E13 8SP UK; 2 Bamford Centre for Mental Health & Wellbeing, University of Ulster, Derry, Northern Ireland; 3 Division of Psychiatry, University College London, Maple House, London, UK; 4 North London Service User Research Forum, Division of Psychiatry, University College London, Maple House, London, UK; 5 Department of Geography, Durham University, Durham, UK; 6 Implementing Recovery through Organisational Change, Mental Health Network NHS Confederation, London, UK

**Keywords:** Supported Accommodation, Mental Health, Effective Practices, Goals, Intentions, Qualitative Study

## Abstract

**Background:**

Deinstitutionalisation in Europe has led to the development of community-based accommodation for people with mental health problems. The type, setting, and intensity of support provided vary and the costs are substantial. Yet, despite the large investment in these services, there is little clarity on their aims and outcomes or how they are regarded by staff and the clients.

**Methods:**

We interviewed 30 staff and 30 clients from the three main types of supported accommodation in England (residential care, supported housing, floating outreach) to explore their perspectives on the purpose of these services, and the components of care considered most helpful. The interviews were coded and analysed using thematic analysis.

**Results:**

There were generally consistent understandings amongst clients and staff across service types on the goals and purposes of supported accommodation services as: building independence and confidence; supporting people with their mental health; and providing safety and stability. We also noted a competing theme of anxiety about the continuity of support when clients move on from a service. Themes on the experience of what aided effective practice centred on: the supportive presence of others; incremental steps to progress; working together to avoid deskilling and dependency; feeling known and personally understood; tailoring support for social and community engagement; and building confidence through encouragement.

**Conclusions:**

The findings provide an understanding of the commonalities in service approach, and goals of clients in these services, as well as the facilitators of goal attainment. However, they also highlight a common tension between providing safe and supportive living environments, whilst also promoting independence and facilitating rehabilitative change.

## Background

The closure of psychiatric hospitals and the integration of patients into communities has stimulated growth in supported accommodation with investment in a variety of residential, semi-supported and independent living situations for people with long-term needs [1, 2] a process viewed by some commentators as ‘re-institutionalisation’ [3, 4]. While the precise models and terminology used to describe supported accommodation varies between countries [2, 5–7], most countries that have undergone deinstitutionalisation provide a range of services with different levels of support at different costs [3, 8]. This paper takes the three most common supported accommodation services in use in the United Kingdom (UK), residential care, supported housing and floating outreach, to explore the aims and experiences of effective practice.

Adequate housing and support with activities of daily living are crucial to maintaining rehabilitation and reducing disability for adults with mental health problems [9–11]. Furthermore, housing problems can contribute to relapse and further hospital admissions [10, 12]. Therefore, accommodation services form an integral component of a whole system rehabilitation pathway for adults with mental health problems [13]. However, less is known about what these services aim to do, and what they actually do in terms of supporting clients to progress [14, 15], with variability noted in service provision, differential improvement in social functioning and low rates of discharge [16, 17]. An understanding is needed on what works for clients in supported accommodation and if all of the goals of these services translate into practice. Whether different models of support actually enable progress towards greater independence.

As part of the rehabilitation pathway in the UK, clients are expected to move through these various forms of supported accommodation. It is estimated that around 60,000 people with complex mental health needs access government funded specialist supported accommodation services in England at significant cost [18–20]. Clients are expected to develop skills and confidence, transitioning from higher support services to lower support, with the eventual aim of managing a tenancy with minimal or no support [13]. Despite their importance and cost to the taxpayer, there is scant evidence about the various forms of supported accommodation and how effective they are in aiding this transition. A recent geographically representative survey of over 150 supported accommodation services in England, indicated few differences between services in client characteristics or in the support provided by staff with personal care and activities of daily living [15].

An in-depth approach is required to understand the identified gap in what service providers and users think residential care, supported housing and floating outreach are meant to be doing, and how effective they are in doing this [21]. If models of supported accommodation have a similar client base and provide similar support, what are the components that enable change and progression to independence? We conducted qualitative interviews to facilitate an understanding of the goals and mechanisms that support change and progress through lived examples. Although not representative of the entire population, a qualitative approach permitted exploration of staff and clients’ potentially differing views on the purpose and experience of supported accommodation services through two related research questions: What are the goals and purpose of supported accommodation services? What factors promote effective practice in supported accommodation services?

## Method

### Setting

This study was conducted in England and included the three main types of mental health supported accommodation: residential care, supported housing and floating outreach services [15]. Residential care homes are staffed 24 h a day, seven days a week, with a high level of support provided, including meals, cleaning, personal care and supervision with medication. Clients share communal facilities and placements tend not to be time-limited, although clients can be supported to transition to more independent settings. Supported housing services can be provided as shared or individual tenancies with staff on-site available most of the day (up to 24 h). Placements are usually time-limited, with services supporting clients to gain skills needed to move to a more independent living situation. Floating outreach services provide staff visiting clients living in time-unlimited independent tenancies. Staff support clients emotionally and assist them to take on more and more responsibility to manage practical tasks (shopping, cooking, cleaning, budgeting, etc.), with the aim of being able to reduce and stop visiting staff support over time [15].

### Sampling and recruitment

The study sample was recruited from clients participating in a national survey of supported accommodation services in England. This qualitative study and the national survey were part of a five year programme of research funded by the National Institute for Health Research (NIHR, RP-PG-0610-10,097) on the Quality and Effectiveness of Supported Tenancies for people with mental health problems (the QuEST study). The services that participated in this survey were selected from a nationally representative sample of 14 Local Authority areas across England. The areas were systematically sampled, based on their ranking on an index score that combined measures of: local mental health morbidity; social deprivation; level of urbanicity; provision of community mental health care; provision of residential care; mental health care spend; and housing demand [22]. Services from each of the three main supported accommodation service types were approached to participate in this qualitative component of the QuEST study by contacting the first service in each area of each service type recruited to the national survey. Where a particular service type was not represented in an area, or the one service of that particular type declined to participate in the survey, we recruited a service from the next area by ascending index rank score. From the 14 areas sampled in the survey, 11 areas spanning the index were represented in the qualitative component. From these 11 areas, 10 staff and 10 clients were recruited from each of three service types to participate in the interviews.

We purposively sampled the 30 clients based on age, gender, service type and length of stay in the service. Service managers provided demographic summarises of the client base and staff within their service, based on this information and knowledge of previously recruited participants to the service type, researchers stratified this information and aimed to recruit subsequent participant types that were not currently represented in the sample. In addition, the 30 staff members were recruited to ensure variation in seniority and experience. We aimed to recruit approximately 15 service managers and 15 support staff from across the three service types.

The researchers aimed to recruit an equal gender split by service type where possible (some services were exclusively, or majority, male or female in composition). Researchers purposively approached 30 staff members to capture variation in seniority and experience, researchers also attempted to recruit approximately half of the staff from the managers in these services, and the remaining half from direct care and support staff.

### Data collection and analysis

The researchers invited staff and clients to be interviewed between October 2013 and July 2014. Potential participants were given an information sheet outlining the study, and were informed that the interview would involve questions about their experiences and views of working or living in supported accommodation services accordingly. Those who verbally agreed to participate were contacted by the researcher to set up the date, time and location (usually a private meeting room in the service, or the client’s room/flat) to gain written consent and to conduct the interview.

Topic guides (for staff and clients) were developed by members of the QuEST project management group (comprising all co-authors), the QuEST service user reference group, and the researchers conducting the interviews (SS, IH, JK & PM). The topic guides covered four areas: i) purpose and aims of the service, ii) positive aspects of the service, iii) negative aspects of the service, and iv) facilitators and barriers to progressing towards more independent living. We explored the experiences, preferences and views of the participants using specific questions; flexible prompts and probes were used to obtain more in-depth responses. Participants were asked to give examples and/or further explain their experiences, significant events and critical issues that could have a bearing on the goals or effectiveness of a supported accommodation service.

The research team aimed to approach data collection and analysis without a priori hypotheses or to test a particular theory. We attempted to cultivate an approach of ‘mindful inquiry’ to note, accept and transcend the influence of our own professional and experiential views and experiences on the interviews process, interpretation and analysis [23]. In terms of methodological approach, we took a critical realist approach to data collection and analysis, viewing participants’ talk as grounded in reality, but with an awareness of the influence of our subjectivity in the interpretation process of the analysis, as well as the social context of the interview [24].

The interviews were transcribed and anonymised prior to being entered into NVivo 10 software [25] for data management and coding. Transcripts were subsequently subjected to inductive semantic thematic analysis [26]. Transcripts were read and re-read for familiarisation. The main research questions formed the basis for the initial coding frame. To capture chronologies, critical events and the comparative perspectives between service types and participant groups, coding was based on the search for patterns, such as similar and different occurrences and processes. We also noted any attributions or explanations for these. Coding was iterative to ensure that important points in the transcripts were recognised and encoded to describe and organise observations prior to interpretation [27, 28]. The transcripts were initially coded by one researcher, with a second checking the consistency and credibility of interpretations for a random selection of 20% of the coded interviews.

Conceptually similar codes were grouped to form categories, which were then grouped to form candidate themes. The analytic process for generating candidate themes was collaborative and involved discussions between the lead investigators (HK, GL and SP) and the researchers (IH, JK, PM and SS) to identify tentative categories and themes that were consistent between clients and staff across the service settings. We used acknowledged guidelines for establishing reliability and validity in qualitative research [29, 30], with potential themes assessed for credibility, applicability, constancy and confirmability in relation to the transcripts and research questions. Interpretation of the themes was monitored by the QuEST project management group and service user reference group. For quality assurance, the conduct of the study and reporting of the results was in line with the consolidated criteria for reporting qualitative research (COREQ) [31]. The finalised thematic structure, with interpretation of the themes and supporting quotations, is presented in the results.

## Results

### Participants

Main participant characteristics by the three service types are presented in Table 1. Thirty clients were interviewed with a mean age of 40 years, slightly more were male (*n* = 17). Client duration of stay in the current supported accommodation service ranged from three months to 14 years, with a mean of three years and four months. Eighteen support workers (60%) and 12 service managers (40%) participated in the staff interviews. Most were female (*n* = 18, 60%), with a mean age of 45 years and a mean duration of working in the supported accommodation service of six and a half years (range of 3 months to 22 years). Staff in all three service types also tended to use the term ‘client’ most frequently in reference to those they supported in their service. For this reason, the reporting of the themes has adopted the term ‘client’ to adequately reflect the consistent terminology in these settings. The duration of the interviews lasted between 16 and 63 min.

**Table 1:** Client, staff and service characteristics by supported accommodation service type

	Mean (s.d.)/n by service type			
Characteristics	Residential Care	Supported Housing	Floating Outreach	All Services
Clients	*n* = 10	*n* = 10	*n* = 10	*n* = 30
Mean age	45.4 (16.3)	33.8 (9.3)	39.8 (13.3)	39.7 (13.7)
Male	6	5	6	17
Female	4	5	4	13
Mean years in current accommodation service	3.7 (3.9)	4.2 (4.5)	2.1 (1.2)	3.3 (3.5)
Range	3 months – 14 years	8 months – 11.5 years	7 months – 4 years	3 months – 14 years
Previous accommodation situation				
Inpatient Rehabilitation Unit	0	2	0	2
Community Rehabilitation Unit	2	0	0	2
Residential Care Home	1	1	0	2
Supported Housing (staffed 24 h)	0	0	3	3
Supported Housing (staffed <24 h)	3	1	2	6
Temporary Accommodation (e.g. bed and breakfast, night shelter/hostel)	1	1	0	2
Rented Property	0	1	3	4
Family Home	3	4	2	9
Staff	*n* = 10	*n* = 10	*n* = 10	*n* = 30
Mean age	52 (11.2)	39.5 (12.5)	43.6 (10.3)	45.0 (12.2)
Male	5	2	5	12
Female	5	8	5	18
Manager/Deputy Manager	5	3	4	12
Support Worker	5	7	6	18
Mean years working in the service	8.2 (8.1)	6.3 (6.4)	4.9 (4.7)	6.5 (6.5)
Range	3 months – 22 years	8 months – 20 years	7 months – 14 years	3 months – 22 years
Service	*n* = 10	*n* = 10	*n* = 10	*n* = 30
Mean number of clients in service	14.4 (5.1)	13.9 (6.9)	29.8 (15.5)	19.4 (12.4)
Mean number of staff in service	15.4 (9.5)	9.6 (9.1)	6.9 (6)	10.6 (8.8)

### The goals and purposes of supported accommodation

Four related themes captured staff and clients’ views of the purpose and goals of supported accommodation services: building independence; building confidence; supporting people with their mental health; and providing safety and stability. However, these themes contrasted with a fifth theme regarding anxiety about the continuity of support when a client moves on from a service. Staff tended to have greater clarity across the service types on what the purpose of the service was for, than the clients did. The frequency of these themes amongst the staff and clients is presented in Table 2, and by service type in Table 3.

**Table 2:** Frequency of themes by participants

	Participants		
Theme	Clients	Staff	Total
*Goals and purposes of support accommodation*	30	30	60
Building independence	24	30	54
Supporting people with their mental health	15	18	33
Providing safety and stability	6	15	22
Building Confidence	9	12	21
Competing theme - Anxiety about the continuity of support	23	18	41
*Helps and aids effective practice in supported accommodation*	30	30	60
The supportive presence of others	25	30	55
Incremental steps	26	29	55
Working together to avoid deskilling and dependency	28	27	55
Feeling known and personally understood	23	26	49
Tailored support for social and community engagement	26	17	43
Building confidence through encouragement	8	27	35

**Table 3:** Frequency of themes by participant’s service type

	Participant’s Service Type		
Theme	Residential Care	Supported Housing	Floating Outreach
*Goals and purposes of support accommodation*	30	30	60
Building independence	19	15	20
Supporting people with their mental health	8	13	12
Providing safety and stability	8	6	8
Building Confidence	8	4	9
Competing theme - Anxiety about the continuity of support	14	12	15
*Helps and aids effective practice in supported accommodation*	30	30	60
The supportive presence of others	20	19	16
Incremental steps	18	19	18
Working together to avoid deskilling and dependency	19	17	19
Feeling known and personally understood	19	13	17
Tailored support for social and community engagement	16	15	12
Building confidence through encouragement	10	12	13

### Building independence

Building the capacity for independent living in the community was the predominant goal discussed by service staff and clients. Supported accommodation staff were viewed as standby support for the client, guiding clients through practical experiences to gain independence, a role differentiated from that of other mental health service staff; where staff are analogous to ‘life coaches’, and the support is metaphorically similar to a ‘crutch’ or ‘stepping stone’.

“We don’t treat people, but we are more like life coaches now. We have kind of….our main role is to help people to maintain their independence, maintain their tenancies, stay in their own home basically.” (manager floating outreach s.01).

“…a place where there’s support, somewhere where I could get back on my feet, which is actually what this place is, you know, based on. It’s like a kind of stepping stone, you know what I mean?” (client residential care s.03)

Staff in all service types acknowledged the limitation of viewing greater independence as equating to a move to more independent accommodation. Thus, building independence was determined by the individual capacity and needs of the client; helping individual clients to progress their lives in a holistic way. Building independence was variously interpreted as clients becoming less reliant on services through the accomplishment of everyday tasks and maintaining their home or, more ambitiously, developing the client’s personal interests and goals beyond accommodation.

“…we’ve got some clients here that we would imagine maybe in two years’ time they’d be able to live either in a minimum support setting; with a view to their moving to independent. Whereas there’s other people here that they would…you know, that is probably not within their reach, for various reasons. So that is… you know, then our job is to work with them to find out what their likes or dislikes are, what they need to [do], to have a good quality of life and independence really.” (manager residential care s.67)

A strongly expressed desire for clients in floating outreach services was the pursuit of ‘normalisation’, that is, to have the same experiences as other people.

“I never thought about it, but it will be great if I became normal like everyone else – could manage everything I want to do, could go like find a job and do some work and feel like everyone else – you know? I wish to one day become like that so I could live independently; I’d really love that – you know? And then become like normal: have a car, have a house or girlfriend or anything you know? Be like everyone else. ” (client floating outreach s.40)

### Building confidence

Another goal of the services was that of building confidence. Clients reported an inability to cope or to do things they once could, often attributed to a lack or loss of confidence. Services were perceived as supporting clients to gain competence in a particular domain in order to help them to build confidence in themselves and their capabilities. Regaining confidence was considered crucial to skill acquisition and a prerequisite to mobilise clients to access their full potential. There was a difference by service type in terms of the importance placed on building confidence as a purpose of the service. Staff and clients in supported housing were less likely to perceive this as a goal of the service (see Table 3) than those in the other service types.

“Well I’m getting my confidence back, ‘cos I haven’t had my confidence for a long while, and I’m getting more respectful towards people and I’m starting to see life in a different way now….” (client residential care s.17)

“I think in the main what it does is it helps develop their feelings of confidence about being able to deal with situations that may not be terrifically familiar, or by which they feel quite threatened, so that they learn how to do it in a more measured way so the confidence comes up, and the next time a similar situation comes around I can say well look, do you remember what we did last time?” (support worker floating outreach s.92)

### Supporting people with their mental health

Staff and clients alike agreed that an integral element of supported accommodation was to promote and support the mental health of the clients through addressing their daily living and social needs. For example, assisting a client with an activity they were concerned about, in order to reduce their anxiety and prevent deteriorating mental health. This view of bolstering mental health was generally couched in social interaction and engagement terms rather than medical intervention. Addressing problems or issues identified in the client’s social environment that impact on their mental health.

“Now, if there are four things that are from a social perspective – if you like, that have an impact on someone’s mental health, if you take those away then you’re looking at…I suppose better mental health. So I would focus on that. The clinical stuff…there are nurses and people and doctors that will look after that element.” (manager floating outreach s.56)

Staff and some clients openly stated that they were living in the supported accommodation because of their mental health and the impact this was having on them personally or people close to them. For other clients, it was only after they had moved into supported accommodation that they realised why they were there, feeling that they had little or no involvement in the decision prior to the move. It was not until they began to receive services that they gained a better understanding of the purpose in terms of specifically supporting them with their mental health and associated problems.

“I still kind of wasn’t very well – mentally I mean – and they said oh, we decided that, you need to go into a Home. So I thought they meant elderly people. So I was sat there like: ‘Do you want me to go into an elderly person’s home?’ Then they said no, the mental health places. So I kind of…because I’d been independent – living with friends and stuff – at first I didn’t really get it, and then obviously I didn’t…wasn’t very happy but when I came here, obviously yeah, I realised that now it was the right decision, and that I did need the support.” (client supported housing s.98)

Staff and clients in residential care were least likely to mention supporting people with their mental health as the purpose of the service, with residential care services more often managing debilitating physical health conditions that required personal care or supervision. In these cases the mental health needs became secondary.

### Providing safety and stability

Providing safety and stability through structured support was regarded as a goal by half of the staff interviewed, and a quarter of the clients. The service was viewed as providing a safe and stable living environment in which to monitor and reduce risks. Thus, both sets of participants acknowledged the need for services to provide emotional reassurance and formal risk management, but staff were considerably more likely to mention safety and stability as a goal of the service than clients were to perceive it as such. The purpose of the service was considered as a ‘safety net’ providing a reliable support network for the client, across all service types.

“I kept like moving around all the time, or like I didn’t…I don’t really get on very well with shared accommodation – and that’s kind of a lot of the accommodation I was in. So it was basically I needed somewhere stable.” (client supported housing s.52)

“…purpose like of this service I think is for safety, security so it’s that basic…that’s what if you ask any of [th]em, that’s what they say straight away – is that they feel safe and secure. And somebody who cares. Basically, well it’s giving them reassurance that, when they do go out – I’m thinking of [*client*], when he does go out, that nobody’s trying to hurt him. Just that reassurance that he will be safe, and if he does have any problems all he’s got to do is come back to me and I will give him the reassurance…” (support worker residential care s.80)

### Anxiety about the continuity of support

A paradoxical consequence of building confidence and creating a safe and secure living environment was the increased likelihood that this would lead to further change. Most participants discussed their anxiety about the uncertainty and potential lack of support when clients move on from a service. Thus, engendering safety and security can also reduce a client’s desire for change. Clients feared changes in their support might harm their progress. Most worried about their ability to cope and manage daily tasks in a new environment. The thought of moving on or severing ties with the service resulted in anxieties about losing their safety net.

“The anxiety’s always been there, it’s not just anxiety it’s things that like I know I wouldn’t cope in a flat on me own…” (client supported housing s.95)

“Yeah that’s because, you know everyone wants to be safe and secure whether, you know whether you’re ill or not, so to have that in the back of your mind to know that you’ve got a place for life, you know is good, but then at the same time, you know you can get caught in a trap and then you think well you know something’s a bit too safe and you don’t wanna move on ‘cos you know you get scared of change.” (client floating outreach s.76)

Confronted by such fears, some staff were often dissuaded from pursuing move-on with clients when they anticipated this response.

“…we’ve tried it once with one person and it was a no-go. As I say I think it’s a safety blanket, and I think it’s a case of not so much us approaching them but them approaching us if they’re ready to move. Because I think if they start to worry and panic and think ‘I’m going to lose me home’…” (manager support housing s.97)

### Helping and aiding effective practice in supported accommodation

Despite the resistance to move on, staff and clients related examples of effective practice in supported accommodation. Six interrelated themes captured these: (i) the supportive presence of others; (ii) incremental steps to progress; (iii) working together to avoid deskilling and dependency; (iv) feeling known and personally understood; (v) tailored support for social and community engagement; and (vi) building confidence through encouragement. The frequency of these themes amongst the staff and clients is presented in Table 2, and by service type in Table 3.

### The supportive presence of others

The support of other staff and residents was considered essential to positive outcomes. Clients reported the presence of others was helpful in terms of feeling safe, reassured, comforted, and afforded the opportunity to engage with others to share similar experiences. When the service promoted respectful interactions between clients this created fertile ground for nurturing protective and personally valued relationships within the service. Clients and staff in floating outreach were least likely to mention the supportive presence of others, as staff would often be required to deliver access to others, or would be the main supportive presence in the client’s life.

“Yeah, I’m not very good at living alone. So just having…if anything goes wrong for me I’ve got, I’ve got the support… yeah residents as well actually. But I’ve lived in supported accommodation because of the having other people around helps really.” (client residential care s.67)

“We might not have actually touched on anything…serious, but just that, just that chin-wag, just that interaction with somebody that’s going through the same thing as you is…it’s just, you can’t put a price on that.” (client supported housing s.69)

“And I think, like I say…just from taking these ladies to the coffee mornings they have…you know it’s not for everybody but, you know you can see that they’ve made friends, they’ve interacted with people, they’ve…you know they’ve shared stories and also I think…it’s empowering really because you can help each other and sort of give each other tips on how you cope with your mental health..” (support worker floating outreach s.71)

However, other clients could also provoked tension and frustration and some people described how the atmosphere created by excessively noisy, inappropriate or disrespectful clients contributed to conflict. Staff frequently used mediation and early intervention methods to deal with tension and resolve conflict between clients in residential care and supported housing. Some clients would also mediate when tension arose between fellow clients. Staff acknowledged the benefit of sharing information and involving others from within the service and professionals from outside to support appropriate decision making.

“..One person hadn’t been taking their medication, was on Clozapine, became quite poorly quite quickly and was quite intimidating to another one of the tenants, and staff intervened pretty quick and basically take them aside, diffuse them…calm the situation down, get in touch with the CPN, and unfortunately that person was admitted to hospital, but sometimes that does happen.” (manager supported housing s.97)

### Incremental steps to progress

Incremental steps were essential to increasing a client’s independence and willingness to move on. This was done through: breaking down concerns into more manageable steps; reducing support hours; moving the client into purpose built less supported ‘stepdown accommodation’ within the service; or continued short-term transitional support after a client had moved to their new accommodation. Essentially, clients viewed services as helpful if they felt they received adequate preparation and support during the process of moving to their new accommodation rather than being abandoned.

“We have a lady and she was really, really poorly when she came to us, very low self-esteem, very anxious, very depressed, very paranoid – wouldn’t eat or drink off the table [stayed in bed]. We’ve taken little steps with her – she is now at the stage where she’s going out on public transport, she’s now going out to activities, she’s now attending appointments on her own, she’s confident in making telephone calls and we think we’ve done really well with her.” (manager residential care s.61)

“It won’t be shared; it’ll be a one-bedroom flat and I will get some support when I move from here, like the safety and support workers that support you for a few months. So they don’t just chuck you out – like you have got something.” (client supported accommodation s.52)

A phased reduction in support helped build a client’s confidence with a skill or activity. Support planning and formal key-working sessions with clients allowed staff to identify goals and assess the potential limitations in achieving them.

“So it’s discussed at every support plan - they happen monthly, two-monthly, three-monthly, depending on what the client’s needs are, so we just discuss about moving on, if it’s moving on from accommodation-based, or whether it’s to another accommodation setting that they require, but we’ll also discuss about the steps towards independent living and what services or support needs to take place to achieve that.” (manager floating outreach s.94)

### Working together to avoid deskilling and dependency

While deskilling and disempowering clients and creating dependency were common concerns irrespective of service type, these negative outcomes could be avoided through joint decision-making in which a process of managed autonomy encouraged partnership working between clients and staff, rather than one of paternalism. Giving advice to clients, prompts, reminders, and sharing distinct parts of an activity were given as examples of this joint effort.

“….what we’re aware of as well is there’s always a chance you can deskill people, and that’s what we’re very aware of; that we don’t wanna bring somebody into support like this and then take away the skills they’ve already learnt by offering too much support if that makes sense – that all your meals are provided here: that’s deskilling somebody…We always try and keep those people, when they come in to us, if they’ve got those skills we don’t want them to lose it.” (support worker supported housing s.19)

“You…you’ve got two sides of the coin, because you’ve got help being offered right? Um, but also you’ve got to help yourself. They go hand-in-hand as it were – if you’re not prepared to help yourself then she’s wasting her time, and that sort of motivates me.” (client floating outreach s.87)

### Feeling known and personally understood

Staff-client trust was essential to help the client feel personally understood and their personhood recognised. This demanded a process of familiarisation and mutual respect, which permitted openness in addressing the client’s needs and risks. This involved questions about client preferences and involving them in decisions. This theme was more commonly mentioned by staff and clients in residential care and floating outreach where proximity and one-to-one time were more readily available to build relationships.

“..she’s aware that I’m intelligent enough to kind of look after myself, but just a little bit sort of overwhelmed with a load of stuff that’s been going on so…and that’s what’s made me fall behind and kind of lose track and things. But from her part kind of using her skills to try and sort of ascertain like what do I need to be doing for this guy and what can he do for himself?....it’s kind of been almost tailor-made for me, which I suspect is sort of a credit to [support worker’s] kind of people skills.” (client floating outreach s.71)

“…a necessary part of relationship building – not just at the beginning, but at times throughout my relationship with clients, to be social. So, maybe suggesting that we go out into town and have coffee - particularly if there’s a level of anxiety about being around a lot of people. And I will do that purposely.” (support worker floating outreach s.92)

### Tailored support and provision for social and community engagement

Social inclusion was also vital to building independence for most participants, but achievable only through a more personalised approach in which social activities were tailored to the client’s needs and tastes. This was done by signposting or, where necessary, actually accompanying the client to an activity, or getting clients to interact more with their local community. Clients mentioned the need for supported social and community engagement more so than the staff. Clients that benefitted the most from the support were the ones that received support to increase or expand pre-existing interests and goals, where support was aligned with the individual’s aspirations and adjusted to their changing needs.

“He’s got me in touch with, I’m not sure – I think they’re a charity and they do all sorts of things, mainly for homeless people but, with mental illness and all sorts and he’s got me in touch with those and I done a course – it was a resilience course; that was for a mental health course and he’s also helped me get onto a bike maintenance course, and also Monday morning down to the canal for fishing…” (client floating outreach s.85)

“We’ve got one gentleman who, he was visiting his step-father every weekend, and he’d done it for the last 20 years, and he turned round to me and said ‘Well I don’t want to go every weekend’ and I’m: what would you rather do? So we sat down at the computer and checked the cinema listings and now every other weekend he goes to the cinema, has a meal with a friend and then comes back. So that we broke out of his routine and he’s really enjoying having a bit more independence.” (manager residential care s.25)

Particularly helpful to clients in floating outreach was regular interaction with their support worker to increase opportunities for any form of social interaction. Staff attributed the lack of opportunities to support clients with social and community engagement to a lack of funding and dedicated time to support this:

“I mean I’m not one to tell people what to do, but I think it would be good for some tenants to get out and do different things, and I think if we were able to do that with tenants, I think the tenants would be amenable to do that. Because a lot of people they don’t want to go out on their own and don’t have that many friends and family, so it’s just like… the social aspect of it and I think it would be beneficial for the tenant’s mental health if we could sort of do a social activity with them. But the supported people contract that we are under, it only allows us to provide housing-related support.” (support worker floating outreach s.76)

### Building confidence through encouragement

Nearly all staff spoke of helping their clients through constant encouragement to engage in activities and actions that would increase their confidence in two ways. First, to demonstrate to the client that they were able to do the activity or engage in the situation they previously felt they were unable to do, or were reluctant to do. Second, staff used knowledge of clients’ past achievements and abilities to encourage new activities.

“Well like I have one client and like he needed a lot of support with his shopping but we sort of go with him in the initial stages or, so we’ll go round with him and then he knows what to buy and then we suggested maybe writing a list out prior with him, he went with the choices that he would use, and then bit by bit we try to drop off our support, so that he can then go by himself and mainly give him lots of encouragement in saying that he’d be fine and he’d manage well on his own, and to try to control his anxiety levels while he was there, and then we only now support if he’s feeling really unwell…but if he’s feeling well enough then we try to encourage him to go by himself.” (manager supported housing s.70)

Clients who appreciated this kind of staff encouragement suggested that it should be linked to a list of requisite practical skills or achievements to give them the reassurance they needed to cope in a more independent living situation.

“Well it’s confidence-building, helping you to deal with things that you need to know about, like it’s how to pay rent and your bills and how to keep yourself clean and…and where you should go.” (client supported housing s.95)

## Discussion

### Main findings

While staff and clients had a shared understanding about the goals and purposes of supported accommodation services, the process of achieving them may be conflicted. Thus, building a secure stable environment may perversely reduce a client’s desire to leave the service. Consequently, staff anticipating a negative reaction or impact on a client may be reluctant to pursue greater independence.

Staff and clients in all three service types shared a similar understand of the facilitators of effective practice. They valued dependable relationships and shared experiences gained between clients and staff, formed through the visible supportive presence and encouragement of others, when clients feel personally known, understood and involved in decisions. To avoid dependency and anxieties about moving on from the service, staff and clients emphasised the need for staff to ‘work with’ clients in incremental ways to achieve progress. Entailed in this was a structure of stepdown procedures from breaking concerns into more manageable steps, reducing support hours gradually, providing transitional support and the use of purpose-built stepdown accommodation. Although important, client involvement in decision-making processes about moving on to more independent accommodation may be limited. We cannot say whether this is due to client passivity, deliberate avoidance to prevent the loss of an established ‘home’ or limited information. All three appear to contribute to client resistance to a move to a more independent living situation. The current rehabilitation system [13] requires the movement of clients through the supported accommodation pathway. However, this may go against the very human need to feel settled and secure, with clients seeking a sense of permanence and familiarity from supported accommodation. The dissonance between the intended supported accommodation pathway and clients actually moving on to more independent living may perpetuate from mental health professionals viewing these services as providing somewhere for clients to live with access to support, while clients may view these placements as more of a ‘home’. This disparity in views has been indicated elsewhere with clients preferring independent accommodation absent of institutional regimes, and professionals preferring environments with staff on-site [32, 33].

In addition, staff and clients in all service types attributed tailored support and provision for social and community engagement as essential for building independence and confidence. For staff and clients in floating outreach services this theme was particularly pertinent. Recognition was given to the often isolated nature is which the clients live with visiting support. The regular interaction with the support staff could often be the main or only opportunity for clients to socially engage with others or access the wider community. In our findings, the floating outreach client group expressed the distinct pursuit for a ‘normal life’, or to have the same experiences as other people. Therefore, limited opportunities for social and community engagement for this group may be particularly pertinent. All staff endorsed the need for more funding and dedicated time to support clients’ preferences for community activities.

### Comparisons to previous literature

The themes identified in this study lend support to those from a review of seven qualitative studies of clients’ accounts in various forms of supported accommodation services in England [21]. Although the review focused primarily on the structural aspects of the housing, it conceptualised three determinants that enabled clients to benefit from the support: autonomy, domain and facilitation. Autonomy was mirrored in our analysis of independence as a goal and path to a ‘normal life’. In Burgoyne’s [21] review it was viewed as a goal and an outcome and was nurtured by environments that empowered clients as individuals with rights and choices. These aspects of empowerment were also presented in our analysis of clients feeling personally known and understood in their own right, which outlined the value placed on choice and involvement in decisions. Burgoyne’s [21] theme ‘Domain’ referred to the physical environment and dwellings themselves, something that was represented less in our findings. However, noise and the negative impact of other clients reflected aspects of our theme on the supportive presence of others through staff mediation and early intervention to counter the potentially negative impacts of shared space. The theme ‘Facilitation’ referred to staff effectively helping clients to live their lives the way they wanted to. This concept mapped onto several of our themes related to effective practice including feeling known and personally understood, providing tailored support for social and community engagement, and building confidence through encouragement.

The most notable difference between our findings and those of Burgoyne’s [21] review was the suggestion that clients in residential care were more likely to report disempowering staff attitudes that would be less likely to be helpful in promoting independence. Our findings did not support this and were more consistent with the view that building independence is a goal across the entire supporting accommodation continuum [34], particularly amongst staff. Furthermore, our findings highlighted some differences between service types, and between staff and clients, absence in this review [21]. Staff tended to be clearer about the goals and purpose of the service, particularly in relation to providing safety and stability. This was a lesser goal of the service from the perspective of the clients. In our findings, for residential care the goal of supporting people with their mental health care would more often compete with physical health goals, mitigating risks for clients with offense histories, and managing deteriorating conditions, compared with those in supported housing and floating outreach. In addition, clients in floating outreach more often reported being the most isolated and in need of support with social and community engagement, whereas residential care and supported housing clients experienced difficulties from being in the presence of others. For staff and clients in floating outreach, a shortage of time and a lack of support from others were limiting factors to building independence and social support.

Our themes also share similarities with recent findings from qualitative studies outside of the UK. Although the precise service models may differ, the variation in level of support is similar. For clients in Sweden and Denmark having a ‘nest’, and forming attachments with others and sharing experiences in supported housing, was akin to the desire for security and the supportive presence of others in our findings [35, 36]. In Australia, when support workers and managers of a floating outreach programme were interviewed about their perceptions of the service, joint effort and involvement to avoid deskilling and dependency was highlighted through the theme of balancing the provision of care with the promotion of autonomy. In addition, feeling known and personally understood was also reflected in the author’s analysis of developing an effective working relationship [37]. Similar to our findings, in the United States of America, qualitative and observational studies with clients in supported accommodation services have noted the importance of stability, security, safety, and a flexible approach to foster a sense of autonomy [38, 39].

Most notably, a review of the international literature on various models of supported accommodation [2] supported the tension we found across service types regarding provision of safety and support whilst promoting independence; in other words, providing a safe and supportive home risks reducing a client’s desire to move on to more independent accommodation. The review concluded that clients generally preferred more independent living situations, but had concerns about with the lack of support and loneliness outside of staffed settings.

### Strengths and limitations

To our knowledge, these findings present the first qualitative analysis of both staff and client views and experiences across the three main types of supported accommodation services in England. Although the precise models may vary between countries, this study represents a range of supported accommodation types broadly familiar in most, in terms of safety, independence and level of staff input. We sought not only to elicit the experiences of those working and living in supported accommodation services, but also to explore the intention or purpose of these services from their perspective. Our analysis was data-led with the direct intention not to propose a particular theory or model on the purpose of supported accommodation. To our awareness, it is the first study to qualitatively analyse the relationship between the two, instead of focusing on one or the other.

However, this is a selective sample and we cannot be sure that we have fully captured the range of views and experiences available in these settings. Staff and clients may have been reluctant to share some of their more negative experiences, or to divulge elements of their practice or behaviours with concerns that they may be perceived or construed in a negative or undesirable way. We have no reason to assume this was the case, but nonetheless we selected participants that had previously been contacted by the researcher and were willing to participant in research. Staff and clients that may have felt apprehensive or marginalised from the services may not have agreed to take part in the national survey from which the potential participants for this study were drawn.

### Implications for practice

The goals and purpose of these services were more clear and consistent amongst staff, regardless of service type. Some work is required with clients to more clearly articulate the purpose of these services, and to bring the clients on board, in order to prevent anxieties about progressing and moving on. The dual purpose of supported accommodation services to provide safe housing as well as supporting rehabilitation presents a common and historic tension in institutional care [3, 40]. However, several of the themes we identified illustrated methods to overcome this including staff and clients working in partnership towards agreed goals in an incremental fashion and being clear about future move-on plans from an early stage in the process. Dedicated time and resources to support these activities are needed from inside and outside of the service. Restricted support hours and isolated services can limit the opportunity for progress. However, sufficient funding and joint working with other agencies and services could reduce this and support significant changes in independence, confidence and safety.

## Conclusions

This study provides an in-depth experiential understanding of the commonalities in service approach for adults with mental health problems in need of supported housing. The goals of clients in these services and the intentions of the staff in these services are similar in terms of: building independence and confidence; supporting people with their mental health; and providing safety and stability. However, there is a common tension between providing safe and supportive living environments whilst also promoting independence and facilitating rehabilitative change. Some of the themes on the experience of what aided effective practice, such as: incremental steps to progress, working together to avoid deskilling and dependency, and tailoring support for social and community engagement provide practical approaches to counter this tension and increase independence to bring about rehabilitative change. These approaches were consistent in the views and experiences of staff and clients alike, and were applicable and acceptable in a range of supported accommodation services, despite the varying levels of support provided.

## Abbreviations

COREQ: Consolidated criteria for Reporting Qualitative research
CPN: Community Psychiatric Nurse
NIHR: National Institute for Health Research
QuEST: Quality and Effectiveness of Supported Tenancies for people with mental health problems
UK: United Kingdom
